# High throughput modular chambers for rapid evaluation of anesthetic sensitivity

**DOI:** 10.1186/1471-2253-6-13

**Published:** 2006-11-10

**Authors:** Yi Sun, Jingqiu Chen, Gregory Pruckmayr, James E Baumgardner, David M Eckmann, Roderic G Eckenhoff, Max B Kelz

**Affiliations:** 1 Department of Anesthesiology and Critical Care, University of Pennsylvania School of Medicine, Philadelphia, USA; 2 Oscillogy LLC, Folsom, PA, USA; 3 Institute for Medicine and Engineering, University of Pennsylvania School of Medicine, Philadelphia, USA; 4 Mahoney Institute for Neurological Science, University of Pennsylvania School of Medicine, Philadelphia, USA

## Abstract

**Background:**

Anesthetic sensitivity is determined by the interaction of multiple genes. Hence, a dissection of genetic contributors would be aided by precise and high throughput behavioral screens. Traditionally, anesthetic phenotyping has addressed only induction of anesthesia, evaluated with dose-response curves, while ignoring potentially important data on emergence from anesthesia.

**Methods:**

We designed and built a controlled environment apparatus to permit rapid phenotyping of twenty-four mice simultaneously. We used the loss of righting reflex to indicate anesthetic-induced unconsciousness. After fitting the data to a sigmoidal dose-response curve with variable slope, we calculated the MAC_LORR _(EC_50_), the Hill coefficient, and the 95% confidence intervals bracketing these values. Upon termination of the anesthetic, Emergence time_RR _was determined and expressed as the mean ± standard error for each inhaled anesthetic.

**Results:**

In agreement with several previously published reports we find that the MAC_LORR _of halothane, isoflurane, and sevoflurane in 8–12 week old C57BL/6J mice is 0.79% (95% confidence interval = 0.78 – 0.79%), 0.91% (95% confidence interval = 0.90 – 0.93%), and 1.96% (95% confidence interval = 1.94 – 1.97%), respectively. Hill coefficients for halothane, isoflurane, and sevoflurane are 24.7 (95% confidence interval = 19.8 – 29.7%), 19.2 (95% confidence interval = 14.0 – 24.3%), and 33.1 (95% confidence interval = 27.3 – 38.8%), respectively. After roughly 2.5 MAC_LORR _• hr exposures, mice take 16.00 ± 1.07, 6.19 ± 0.32, and 2.15 ± 0.12 minutes to emerge from halothane, isoflurane, and sevoflurane, respectively.

**Conclusion:**

This system enabled assessment of inhaled anesthetic responsiveness with a higher precision than that previously reported. It is broadly adaptable for delivering an inhaled therapeutic (or toxin) to a population while monitoring its vital signs, motor reflexes, and providing precise control over environmental conditions. This system is also amenable to full automation. Data presented in this manuscript prove the utility of the controlled environment chambers and should allow for subsequent phenotyping of mice with targeted mutations that are expected to alter sensitivity to induction or emergence from anesthesia.

## Background

The mechanisms that give rise to anesthetic-induced unconsciousness have eluded understanding for more than 150 years. Breakthroughs in circadian timekeeping and sleep neurobiology have been made in recent years using genetic approaches to unravel mutant phenotypes in mice [1-4]. Genetic approaches offer an unparalleled opportunity to elucidate complex biological problems such as anesthetic action. One characteristic of successful behavioral genetic endeavors has been the development of rapid screens to identify mutant phenotypes [5]. A common feature of these rapid screens is that they have motor-dependent endpoints. Anesthetic-induced unconsciousness is typically assessed using a loss of righting reflex (LORR) assay. As with wheel running, this endpoint depends upon movement and is amenable to full automation – another hallmark of successful forward genetic screens [5]. The LORR assay has been used with mice to identify genetic mutations that cause hypersensitivity [6,7] or resistance [8] to various anesthetic agents.

In humans, general anesthesia is typically separated into three phases: induction, maintenance, and emergence. Animal studies of anesthetic sensitivity have failed to recognize these three phases, likely due to the inherent difficulty in maintaining intravenous or intraperitoneal steady-state levels of typical anesthetic agents such as propofol, etomidate, barbiturates, or ketamine. However, with inhaled anesthetics, steady-state drug levels are easily achieved. Multiple studies have attempted to decipher molecular mechanisms crucial for induction of anesthesia (reviewed in [9-12]), yet only rare reports characterize mechanistic changes that occur upon emergence from anesthesia [13-16]. Many clinical case reports suggest that a variety of conditions can modulate emergence from anesthesia with the most common finding being delayed emergence in specific subsets of patients [17-21]. There is a paucity of animal data investigating pharmacologic treatments which might modify emergence [22,23]. To date, there is only a single genetics study investigating particular phenotypes of mice that exhibit altered emergence patterns following general anesthetics [24]. Greater insight into the mechanisms through which anesthetics induce unconsciousness as well as the mechanisms leading to its return are necessary to gain understanding of the entire process of general anesthesia.

In this study we report the novel design and testing of high throughput controlled environment chambers that will allow detailed phenotypic characterization of murine sensitivity to induction as well as emergence from general anesthesia. This apparatus should facilitate both unbiased forward genetic screens and putative anesthetic target reverse genetic screens to evaluate molecular mechanisms of inhaled anesthetic action. We have taken great care to control for known confounders of anesthetic sensitivity such as body temperature, circadian time, and anxiety of mice in an unfamiliar testing environment. Using these chambers we have studied the wild type sensitivity to induction and emergence from 3 volatile anesthetics in C57BL/6J male mice, aged 8–12 weeks.

## Methods

**Figure 1:**
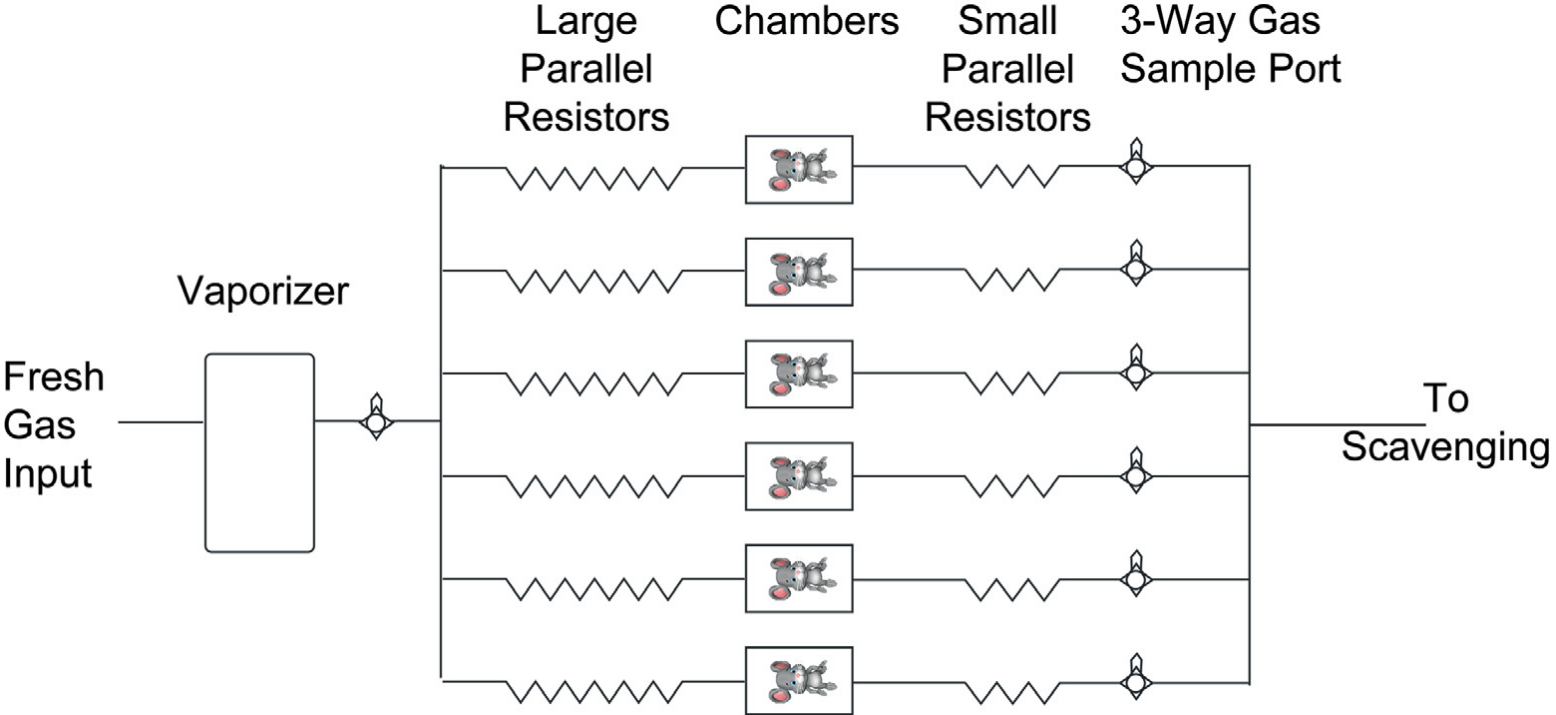
**Schematic Illustration of controlled environment chambers**. For the purposes of this diagram only six modules depicting six mice are shown. For actual experiments, twenty-four controlled environment chambers were arrayed in parallel as shown above. Note that the limit for this design is based only on space and vaporizer performance. Recently capacity was increased to thirty chambers, which still exhibited identical gas flows thanks to the effects of each high resistance element placed upstream of each individual chamber.

**Figure 3:**
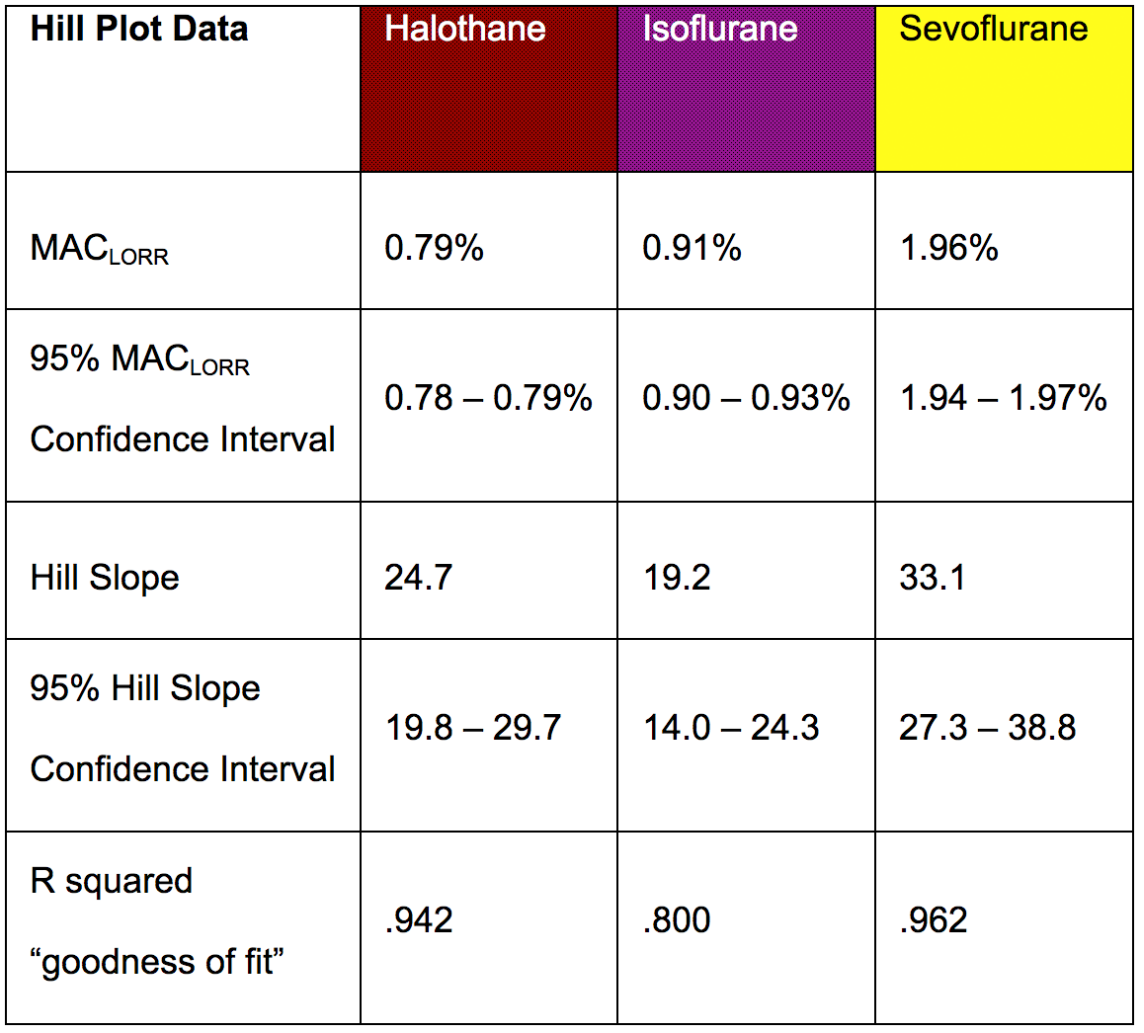
**Statistical analysis of MAC_LORR _dose-response curves**. Data were fit with a logistic equation of the form: Y = X^Hill slope^/[X^Hill slope ^+ MAC_LORR_^Hill slope^] where Y is the fraction of the population that has lost its righting reflex, X is the anesthetic concentration, MAC_LORR _is the dose at which 50% of the population has lost its righting reflex, and Hill slope is a constant that represents the steepness of the dose-response curve. The accompanying table reports best fit values for MAC_LORR _and Hill slope for each anesthetic gas along with the 95% confidence intervals surrounding the fit.

Male C57BL/6J mice aged 8–12 week old weighing 23.6 ± 0.4 g (Jackson Laboratories, Bar Harbor, ME) were used in this study, which was approved by the Animal Care and Use Committee at the University of Pennsylvania. Mice were maintained on a 12-hour light/dark cycle and had *ad libitum *access to water and rodent chow before each day's experiments. We built an open-circuit rodent anesthetizing system capable of simultaneously holding twenty-four mice in isolated chambers provided with identical microenvironments. Each of the 24 cylindrical chambers had an internal diameter of 2.25 inches, a length of 3.875 inches, and a volume of 250 ml. On each experimental day, inlet gas pressure upstream of the parallel resistors was 2.5 PSI (above atmospheric) which resulted in a uniform flow 108.0 ± 4.3 ml/min (figure 1 and tables 1, 2) as determined by a mass flowmeter (Omega Engineering, Stamford, CT). Groups of 24 mice were habituated to the Loss of Righting Chambers for two hours each day on four successive days. During habituation, all mice breathed 100% oxygen. Carbon dioxide levels were measured during habituation sessions in the individual chamber exhaust with a Datex Capnomatic II gas analyzer (Helsinki, Finland). Temperature studies demonstrated that a constant body temperature could be maintained by immersing the entire anesthetizing apparatus in a water bath (table 3). Submersion also allowed a daily gas leak test in the chambers and associated tubing. Induction of general anesthesia was accomplished with Drager model 19.1 isoflurane, halothane, or sevoflurane vaporizers using eight to ten stepwise incremental increases in the concentration of anesthetic gas in oxygen. Initial concentrations of volatile anesthetic gases were halothane 0.62% ± 0.02%, isoflurane 0.67% ± 0.06%, and sevoflurane 1.56% ± 0.04%. After 15 minutes at each concentration to allow equilibration of the mouse with the anesthetic vapors in the chamber, the concentration of volatile anesthetic was increased by 5 ± 1% of the preceding value. Peak concentrations of volatile anesthetic gases were halothane 0.93% ± 0.01%, isoflurane 1.07% ± 0.07%, and sevoflurane 2.22% ± 0.01%. Anesthetic gas concentration was determined in real time to two decimal point accuracy with three independent samples analyzed in the last two minutes of each 15-minute interval on a Riken FI-21 refractometer (AM Bickford, Wales Center, NY) in 100% oxygen carrier gas mode. Pilot studies of gas flow indicated that each of the twenty-four chambers reached equilibrium within the first 6 minutes, leaving 9 minutes for the average 25 g mouse with a minute ventilation of 30 ml/min and a cardiac output of 12–20 ml/min [25,26] to equilibrate in the chamber. At the end of each fifteen-minute interval, the cylindrical chambers were rotated 180 degrees. A mouse was considered to have lost the righting reflex if it was unable to turn itself prone onto all four of its feet in the ensuing two minutes. After the last of the twenty-four mice had lost its righting reflex, volatile anesthetic concentration was increased one additional time prior to measurements of emergence time_RR_. With all mice in the supine position, the anesthetic vapors were shut off (time = 0 seconds) and the duration of time that elapsed until each mouse regained its righting reflex by turning prone onto all four feet was recorded. The total anesthetic dose, defined as the integral of anesthetic gas concentration multiplied by time at that concentration (15 minutes) divided by the MAC_LORR _for each agent as determined (see text below and figure 3), was defined as the MAC_LORR _• hr and was recorded. After determining that mouse body temperature was held within 0.6°C of baseline in a pilot group of animals when the last mouse had lost its righting reflex (rectal temperature probe, Physitemp Clifton, NJ), rectal temperature was verified in each mouse once the last animal had emerged from anesthesia. All anesthetic gases were actively scavenged with wall suction. A room air pop-off valve maintained the twenty-four mouse anesthetizing chambers at 1 atmosphere.

**Table 1:** Flow data (ml/min) as a function of 4 different input pressures

Input Pressure Setting (PSI)	**A1**	**A2**	**A3**	**A4**	**A5**	**A6**	**B1**	**B2**	**B3**	**B4**	**B5**	**B6**	**C1**	**C2**	**C3**	**C4**	**C5**	**C6**	**D1**	**D2**	**D3**	**D4**	**D5**	**D6**
2.0	88	86	78	77	79	88	88	88	89	79	89	88	87	88	89	89	87	88	89	89	89	88	83	87
2.5	110	109	99	98	100	110	110	110	112	99	111	110	109	109	110	110	108	108	110	110	110	109	102	108
5.0	165	163	148	147	151	167	165	165	168	150	167	167	165	167	168	169	165	167	168	168	168	168	157	166
7.5	216	213	195	193	198	217	217	217	220	197	219	218	216	217	218	218	214	216	218	219	218	217	202	215

**Table 2:** Flow data (percent total flow) as a function of 4 different input pressures.

Input Pressure Setting (PSI)	**A1**	**A2**	**A3**	**A4**	**A5**	**A6**	**B1**	**B2**	**B3**	**B4**	**B5**	**B6**	**C1**	**C2**	**C3**	**C4**	**C5**	**C6**	**D1**	**D2**	**D3**	**D4**	**D5**	**D6**
2.0	4.25	4.15	3.77	3.72	3.82	4.25	4.25	4.25	4.30	3.82	4.30	4.25	4.20	4.25	4.30	4.30	4.20	4.25	4.30	4.30	4.30	4.25	4.01	4.20
2.5	4.26	4.22	3.84	3.80	3.87	4.26	4.26	4.26	4.34	3.84	4.30	4.26	4.22	4.22	4.26	4.26	4.18	4.18	4.26	4.26	4.26	4.22	3.95	4.18
5.0	4.21	4.16	3.78	3.75	3.85	4.26	4.21	4.21	4.29	3.83	4.26	4.26	4.21	4.26	4.29	4.31	4.21	4.26	4.29	4.29	4.29	4.29	4.01	4.24
7.5	4.23	4.17	3.82	3.78	3.88	4.25	4.25	4.25	4.31	3.86	4.29	4.27	4.23	4.25	4.27	4.27	4.19	4.23	4.27	4.29	4.27	4.25	3.95	4.21
**%Avg**	4.24	4.18	3.80	3.76	3.86	4.26	4.24	4.24	4.31	3.83	4.29	4.26	4.22	4.25	4.28	4.29	4.20	4.23	4.28	4.28	4.28	4.25	3.98	4.21

In this table, data from Table 1 have been transformed and are expressed as a percentage of total flow in each of the 24 chambers. The average flow per chamber independent of input pressure is displayed in the last row. Each of the individual 24 chambers was found to receive 4.17 ± 0.17 % of the total flow (mean ± standard deviation)

**Table 3:** Anesthetic Effects upon Mouse Body Temperature

**Condition**	**Average**	**Standard Error**	**Number of Mice**
Baseline Temperature Prior to placement in chambers (FGF = 0) No anesthetic exposure No active warming	37.3	0.1	109
Temperature after 2 hr exposure to FGF = 100 ml/min oxygen No anesthetic exposure No active warming	36.6	0.1	24
Temperature after 2 hr exposure to FGF = 100 ml/min oxygen Anesthetic = Isoflurane 1.25% No active warming	25.4	2.3	30
Temperature after 2 hr exposure to FGF = 100 ml/min oxygen Anesthetic = Isoflurane 1.25% Apparatus in water bath set to 33°C	33.0	0.2	10
Temperature after 2 hr exposure to FGF = 100 ml/min oxygen Anesthetic = Isoflurane 1.25% Apparatus in water bath set to 36°C	36.2	0.2	10
Temperature after 2 hr exposure to FGF = 100 ml/min oxygen Anesthetic = Isoflurane 1.25% Apparatus in water bath set to 40°C	39.8	0.2	11
Temperature after 2 hr exposure to FGF = 100 ml/min oxygen No Anesthetic Exposure Apparatus in water bath set to 37°C	36.5	0.1	24
Temperature after 2 hr exposure to FGF = 100 ml/min oxygen Anesthetic = Isoflurane 1.25% Apparatus in water bath set to 37°C	36.6	0.1	16

Temperature measurements at baseline and following 2 hours of exposure to 100 ml/min oxygen fresh gas flow (FGF) in controlled environment chambers (250 ml volume apiece) in the presence or absence of 1.25% isoflurane and varying degrees of warming obtained by submerging controlled environment chambers in a water bath. Room temperature varied between 20–22°C.

### Statistical analysis

To obtain MAC_LORR _and Hill slope constants, plots of the log of the volatile anesthetic gas concentration versus %population having lost the righting reflex were generated and fit with a non-linear dose-response curve with a variable slope using Prism 4.0 (GraphPad Software, Inc., San Diego, CA). In order to minimize the number of animals used in the study and to confirm the reproducibility of the data generated, a population of twenty-four mice was exposed to the same anesthetic four times. Mice had a minimum of 24 and a maximum of 72 hours between anesthetic exposures. MAC_LORR _and Hill slope constants are reported as mean of four independent trials with 95% confidence limits. Emergence time_RR _data generated immediately following each anesthetic exposure is reported as a mean ± standard error.

## Results

Prior to putting mice in the modular anesthetizing chambers, we verified the parallel circuit design by confirming that each of the 24 chambers received equivalent gas flow, Tables 1 and 2. By placing an anesthetic vaporizer upstream of the parallel branching, identical concentrations of volatile anesthetics were delivered to each of the 24 chambers. The anesthetizing circuit was an open design predicted to have laminar flow based upon a calculated Reynolds number of 2.8. With an average of 108 ml/min fresh gas flow applied to each chamber, expired CO_2 _was measured from each mouse by sampling individual chamber outflow gases. The maximal CO_2 _measurement ever obtained was 14 torr (1.8%) upon the first entry of a mouse into its chamber. The average CO_2 _outflow measurement was 8.4 ± 0.5 torr on the first day of habituation. Over the course of 4 successive days, CO_2 _levels in chamber egress gas dropped from 8.4 ± 0.5 torr to 4.8 ± 0.1 torr. This suggests that CO_2 _production decreased as the mice become accustomed to the confined chamber, figure 1.

When placed in the chambers and exposed to 100 ml/min fresh gas flow, unanesthetized mice drop their core body temperature from 37.3 ± 0.1°C to 36.6 ± 0.1°C. Without active heat transfer, mice exposed to 1.25% isoflurane in dehumidified oxygen for two hours were found to cool to 25.4 ± 0.4°C, which approaches room temperature (table 3). Since sensitivity to anesthesia is significantly modulated by temperature, we designed a means of conductive heat transfer by placing the chambers in a heated water bath. With the water bath set to 37°C, we were able maintain mice at 36.6 ± 0.1°C, eliminating hypothermia as a confounder regardless of the duration of the anesthetic exposure.

**Figure 2:**
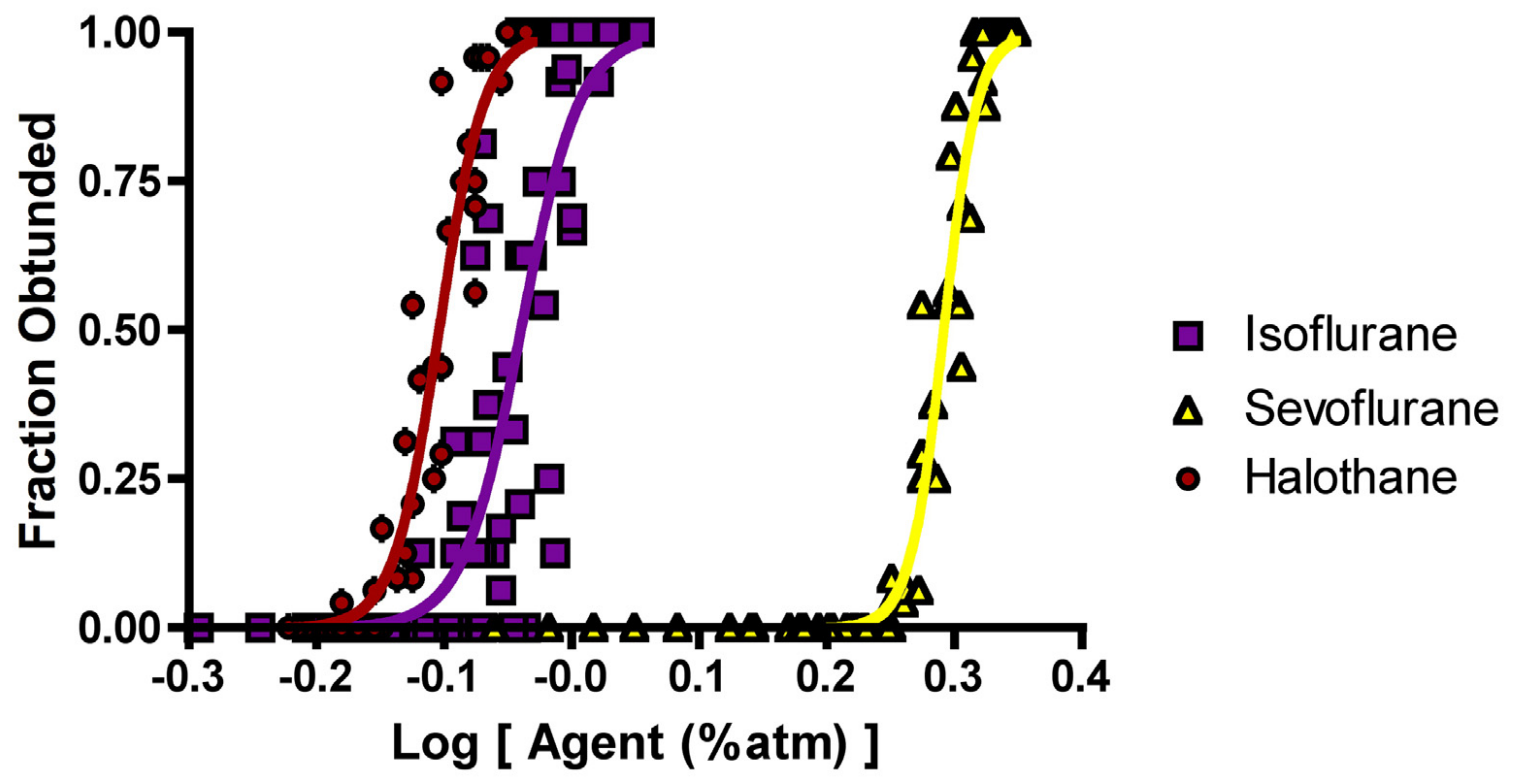
**MAC_LORR _dose-response curves**. MAC_LORR _Dose-Response Curves displayed on a standard semi-log plot demonstrating responses of a population of 24 wild type C57BL/6J male mice aged 8–12 weeks to increasing doses of halothane (red circles), isoflurane (purple squares), and sevoflurane (yellow triangles).

**Figure 4:**
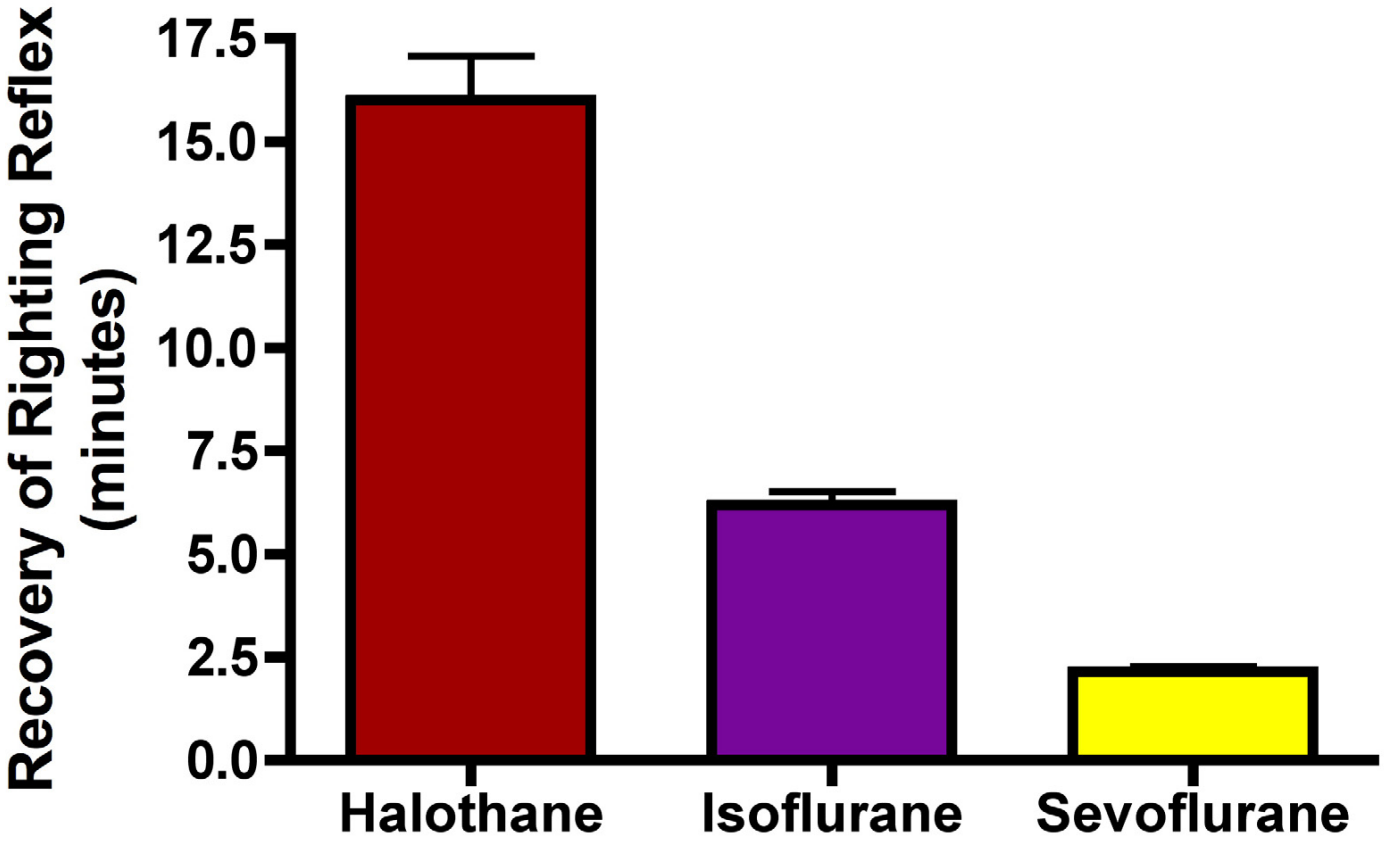
**Emergence Times_RR _for Halothane, Isoflurane, and Sevoflurane**. Following the stepwise increases in anesthetic gas concentration, anesthetic gases were abruptly discontinued after the last mouse had lost its righting reflex. The duration of time that elapsed until all mice regained their righting reflexes was recorded. Data are displayed as the mean ± standard error. The cumulative anesthetic doses were 2.4 ± 0.1, 2.8 ± 0.1, and 2.7 ± 0.3 MAC_LORR _• hrs for halothane (red), isoflurane (purple), and sevoflurane (yellow) respectively.

Having optimized the anesthetizing chambers with appropriate environmental controls (temperature, humidity, and inhaled gas composition) we subjected mice to MAC_LORR _determinations as described in the methods. Figure 2 shows the population response for wild type C57BL/6J mice exposed to halothane, isoflurane, and sevoflurane. Best-fit parameters for data are displayed in figure 3. Emergence from anesthesia as defined by recovery of the righting reflex is shown in figure 4. Cumulative anesthetic doses prior to emergence were 2.4 ± 0.1, 2.8 ± 0.1, and 2.7 ± 0.3 MAC_LORR _• hrs for halothane, isoflurane, and sevoflurane respectively.

## Discussion

We have constructed and validated modular chambers to conduct high throughput screens of anesthetic-induced loss of righting reflex and its subsequent return. Using inhaled anesthetic gases, we studied induction and emergence from anesthesia by measuring the following parameters: 1) MAC_LORR_, defined as the minimum alveolar concentration at which 50% of the test population has lost its righting reflex; 2) Hill slope which evaluates the steepness of the transition from wakefulness to general anesthesia for the test population; and 3) emergence time_RR_, defined as the duration of time before a population regains its righting reflex. Whereas most studies of anesthetic sensitivity evaluate MAC (MAC_immobility_), defined as the minimum alveolar concentration required to prevent movement in 50% of a population in response to a noxious stimulus such as a tail pinch [27-29], we are primarily interested in the hypnotic component of anesthesia. As outlined by Mashour and colleagues, the hypnotic component of anesthesia is most precisely defined as a lack of "perceptive awareness" [10] and should therefore be evaluated following a non-noxious stimulus such as turning an animal prone to supine to examine its righting reflex. The righting reflex provides one behavioral measure commonly used to assess hypnosis in rodents. However, when used alone this assay could yield false positive interpretations of behavioral state. If one administered a muscle relaxant by itself, treated mice would exhibit LORR while remaining fully aware. Hence, for a formal genetic analysis, MAC_LORR _and emergence time_RR _data should be combined with a second index of behavioral state such as EEG-based analyses. An EEG-based secondary screen would reveal cortical signatures of anesthetic action that should compliment the righting reflex's brainstem signatures [30].

Our reported values for MAC_LORR _are in good agreement with values obtained by Homanics and colleagues using a similar best-fit approach for sigmoidal dose response data [31]. The small difference in MAC_LORR _for halothane (our determination is 13% greater than theirs) is easily accounted by the different genetic background of their mice which could influence anesthetic sensitivity by as much as 40% [32]. Our best fit values for Hill coefficients are within the expected range for volatile anesthetics of 20–30 [33]. Lastly, the "goodness of fit" for our behavioral data as measured by r^2 ^obtained with our apparatus is high relative to the literature (figure 3). We attribute these high best-fit values to reductions in experimental error both due to the uniform delivery of experimental conditions among all mice in our controlled environment anesthetizing chambers on a given day and to the reproducibility of conditions achieved with our chambers on different days.

Several other groups have sought to determine MAC_LORR _in mice. While attempting to assess the hypnotic component of anesthesia in mice with mutations in the AMPA receptor, GluR2 [6], and also in the nicotinic acetylcholine receptor subunit, beta 2 [34], lower values for MAC_LORR _were reported. Joo and colleagues quoted MAC_LORR _for isoflurane at 0.57 ± 0.16%, and sevoflurane at 1.16 ± 0.19% in their wild type controls. Their range for halothane of 0.67 ± 0.20% was similar to ours. Also in agreement with Joo's MAC_LORR _findings are those of Flood and colleagues [34] who found MAC_LORR _of isoflurane in inbred 129 mice was 0.56 ± 0.10% and MAC_LORR _of isoflurane in C57BL/6J mice was 0.63 ± 0.20%. One major methodological difference could account for the discrepancy in MAC_LORR_. Both Joo and Flood used a bracketing measurement, which averages the highest anesthetic concentration at which mice still retain the righting reflex with the lowest anesthetic concentration at which mice have lost the righting reflex to estimate MAC_LORR_. If the incremental anesthetic concentration step size is large, systematic error in MAC_LORR _determination can't be avoided, as Hill slopes for anesthetic drugs are notoriously steep.

In creating our modular, high throughput, controlled environment, anesthetizing chambers, we were confronted with several significant design problems. The first revolved around the high metabolic rate of mice. With a minute ventilation of approximately 30 ml/min [26] and a carbon dioxide production rate, V_CO2_, of 1.3 ml/min at rest [35,36], any arrangement of mouse chambers in series would lead to rebreathing at low fresh gas flows, evaporative and convective heat loss at high fresh gas flows, and heterogeneous conditions for mice at different positions relative to the fresh gas inlet. Thus, it became clear that the mice must be arrayed in parallel. Even with each animal having its own fresh gas source, carbon dioxide handling issues remained. We reasoned that during initial exposure to the chambers, mice would experience a mild restraint stress caused by confinement in the 250 ml tubes (figure 1). This would increase carbon dioxide production. With repeated exposure during 4 daily habituation sessions, measured expired carbon dioxide levels from all mice decreased, consistent with a decrease in V_CO2 _production during habituation. Further, we reasoned that at 100 ml/min fresh gas flow in each chamber, convective flow should vastly exceed the capacity of CO_2_-rich expired gases to back diffuse into the inspired fresh gas flow, thus reducing rebreathing. Nonetheless, even assuming the worst-case scenario of perfect mixing in each chamber, and a maximal murine V_CO2 _of 100 ml·kg^-1^·min^-1^, the theoretical upper limit for inspired CO_2 _in our chambers for a 25 g mouse receiving fresh gas flow at 100 ml/min is 2.5%. Our data suggests that the mice only produce enough CO_2 _to occupy up to 1.1% on initial entry in the chambers on day one of habituation and that this declines to 0.6% on the last day of habituation. It should be noted that these values represent the maximum theoretical upper limit for inspired CO_2_. Actual inspired CO_2 _concentrations will be much lower as net fresh gas flow will be laminar across any given chamber and convective flow should predominate.

Another challenge posed by this open circuit setup of our chambers is the enormous potential for convective heat loss in anesthetized mice. Unanesthetized mice, capable of normal thermoregulation, experienced only a small decline in body temperature (0.6°C), which was not significantly different if the chambers were submerged in a 37°C water bath. While the awake mouse maintains its core body temperature through vasoconstriction, anesthetic gases inhibit central and peripheral thermoregulation [37]. Therefore, in the anesthetized mouse, heat transfer to the chambers submerged in the water bath is essential to maintain normothermia.

Whereas we have chosen to use these controlled environment chambers to deliver an inhaled anesthetic and measure hypnotic responses of wild type mice, this system could easily be used to deliver any inhaled therapeutic or toxin in any setting where precise control over the testing environment is necessary. With our parallel open circuit design, gas flow is evenly divided between the 24 individual chambers. However, there is no reason why the number of chambers is limited to 24. We have recently increased the capacity to 30 chambers while still maintaining identical individual microenvironments. Gas composition, humidity, and test subjects' temperatures can be easily regulated. Moreover, by implanting telemetric transmitters in mice, it is possible to stream other physiologic data from each of the 24 pods to corresponding detectors. In this way, vital signs such as blood pressure, heart rate, respiratory rate, core body temperature, as well as electroencephalographic and electromyographic signals that may be altered by the delivery of an inhaled therapeutic can be continuously monitored. We believe that development of apparatus along these lines will enhance our ability to rapidly dissect the pharmacogenetics of volatile drug action.

## Conclusion

This study lays the foundation for rapid screening of genes that alter anesthetic sensitivity in mice by establishing a reproducible, high throughput system for the analysis of anesthetic-induced loss and subsequent recovery of the righting reflex, a commonly used behavioral correlate of anesthetic-induced unconsciousness in rodents.

## List of abbreviations

AMPA: alpha-amino-3-hydroxy-5-methyl-4-isoxazolepropionic acid

Emergence time_RR_: duration of time from discontinuation of anesthetic until the righting reflex returns

FGF: fresh gas flow

LORR: loss of righting reflex

MAC_LORR_: inspired concentration of inhaled anesthetic at which 50% of population looses their righting reflex

PSI: pounds per square inch

V_CO2_: rate of carbon dioxide production

## Competing interests

The authors are in the process of applying for a patent covering the design of the controlled environment chambers described in this manuscript.

## Authors' contributions

YS, JC, GP all carried out the behavioral experiments described in the manuscript. JEB created the initial design of the controlled environment chambers. RGE, and DME all made essential modifications to the design of the controlled environment chambers. MBK conceived the project, performed the statistical analysis, and wrote the manuscript. All authors read and approved the final manuscript.

## Pre-publication history

The pre-publication history for this paper can be accessed here:



## References

[B1] King DP, Takahashi JS (1996). Forward genetic approaches to circadian clocks in mice. Cold Spring Harb Symp Quant Biol.

[B2] Antoch MP, Song EJ, Chang AM, Vitaterna MH, Zhao Y, Wilsbacher LD, Sangoram AM, King DP, Pinto LH, Takahashi JS (1997). Functional identification of the mouse circadian Clock gene by transgenic BAC rescue. Cell.

[B3] Lin L, Faraco J, Li R, Kadotani H, Rogers W, Lin X, Qiu X, de Jong PJ, Nishino S, Mignot E (1999). The sleep disorder canine narcolepsy is caused by a mutation in the hypocretin (orexin) receptor 2 gene. Cell.

[B4] Sakurai T, Amemiya A, Ishii M, Matsuzaki I, Chemelli RM, Tanaka H, Williams SC, Richarson JA, Kozlowski GP, Wilson S, Arch JR, Buckingham RE, Haynes AC, Carr SA, Annan RS, McNulty DE, Liu WS, Terrett JA, Elshourbagy NA, Bergsma DJ, Yanagisawa M (1998). Orexins and orexin receptors: a family of hypothalamic neuropeptides and G protein-coupled receptors that regulate feeding behavior. Cell.

[B5] Bucan M, Abel T (2002). The mouse: genetics meets behaviour. Nat Rev Genet.

[B6] Joo DT, Gong D, Sonner JM, Jia Z, MacDonald JF, Eger EI, Orser BA (2001). Blockade of AMPA receptors and volatile anesthetics: reduced anesthetic requirements in GluR2 null mutant mice for loss of the righting reflex and antinociception but not minimum alveolar concentration. Anesthesiology.

[B7] Simpson VJ, Rikke BA, Costello JM, Corley R, Johnson TE (1998). Identification of a genetic region in mice that specifies sensitivity to propofol. Anesthesiology.

[B8] Jurd R, Arras M, Lambert S, Drexler B, Siegwart R, Crestani F, Zaugg M, Vogt KE, Ledermann B, Antkowiak B, Rudolph U (2003). General anesthetic actions in vivo strongly attenuated by a point mutation in the GABA(A) receptor beta3 subunit. Faseb J.

[B9] Campagna JA, Miller KW, Forman SA (2003). Mechanisms of actions of inhaled anesthetics. N Engl J Med.

[B10] Mashour GA, Forman SA, Campagna JA (2005). Mechanisms of general anesthesia: from molecules to mind. Best Pract Res Clin Anaesthesiol.

[B11] Hemmings HC, Akabas MH, Goldstein PA, Trudell JR, Orser BA, Harrison NL (2005). Emerging molecular mechanisms of general anesthetic action. Trends Pharmacol Sci.

[B12] Rudolph U, Antkowiak B (2004). Molecular and neuronal substrates for general anaesthetics. Nat Rev Neurosci.

[B13] Rentero N, Bruandet N, Viale JP, Quintin L (1998). Catechol activation in the vasomotor center upon emergence from anesthesia: specificity. Synapse.

[B14] Bruandet N, Rentero N, Debeer L, Quintin L (1998). Catecholamine activation in the vasomotor center on emergence from anesthesia: the effects of alpha2 agonists. Anesth Analg.

[B15] Anzawa N, Kushikata T, Ohkawa H, Yoshida H, Kubota T, Matsuki A (2001). Increased noradrenaline release from rat preoptic area during and after sevoflurane and isoflurane anesthesia. Can J Anaesth.

[B16] Ohkawa H, Kushikata T, Satoh T, Hirota K, Ishihara H, Matsuki A (1995). Posterior hypothalamic noradrenaline release during emergence from sevoflurane anesthesia in rats. Anesth Analg.

[B17] Gan TJ, Glass PS, Sigl J, Sebel P, Payne F, Rosow C, Embree P (1999). Women emerge from general anesthesia with propofol/alfentanil/nitrous oxide faster than men. Anesthesiology.

[B18] Mesa A, Diaz AP, Frosth M (2000). Narcolepsy and anesthesia. Anesthesiology.

[B19] Crowe S, McKeating K (2002). Delayed emergence and St. John's wort. Anesthesiology.

[B20] Bouw J, Leendertse K, Tijssen MA, Dzoljic M (2003). Stiff person syndrome and anesthesia: case report. Anesth Analg.

[B21] Nakazawa K, Yamamoto M, Murai K, Ishikawa S, Uchida T, Makita K (2005). Delayed emergence from anesthesia resulting from cerebellar hemorrhage during cervical spine surgery. Anesth Analg.

[B22] Meuret P, Backman SB, Bonhomme V, Plourde G, Fiset P (2000). Physostigmine reverses propofol-induced unconsciousness and attenuation of the auditory steady state response and bispectral index in human volunteers. Anesthesiology.

[B23] Plourde G, Chartrand D, Fiset P, Font S, Backman SB (2003). Antagonism of sevoflurane anaesthesia by physostigmine: effects on the auditory steady-state response and bispectral index. Br J Anaesth.

[B24] Lowes DA, Galley HF, Lowe PR, Rikke BA, Johnson TE, Webster NR (2005). A microarray analysis of potential genes underlying the neurosensitivity of mice to propofol. Anesth Analg.

[B25] Kass DA, Hare JM, Georgakopoulos D (1998). Murine cardiac function: a cautionary tail. Circ Res.

[B26] Lindstedt L, Schaeffer PJ (2002). Use of allometry in predicting anatomical and physiological parameters of mammals. Lab Anim.

[B27] Quasha AL, Eger EI, Tinker JH (1980). Determination and applications of MAC. Anesthesiology.

[B28] Eger EI (2001). Age, minimum alveolar anesthetic concentration, and minimum alveolar anesthetic concentration-awake. Anesth Analg.

[B29] Sonner JM, Antognini JF, Dutton RC, Flood P, Gray AT, Harris RA, Homanics GE, Kendig J, Orser B, Raines DE, Trudell J, Vissel B, Eger EI (2003). Inhaled anesthetics and immobility: mechanisms, mysteries, and minimum alveolar anesthetic concentration. Anesth Analg.

[B30] Bignall KE (1974). Ontogeny of levels of neural organization: the righting reflex as a model. Exp Neurol.

[B31] Homanics GE, Ferguson C, Quinlan JJ, Daggett J, Snyder K, Lagenaur C, Mi ZP, Wang XH, Grayson DR, Firestone LL (1997). Gene knockout of the alpha6 subunit of the gamma-aminobutyric acid type A receptor: lack of effect on responses to ethanol, pentobarbital, and general anesthetics. Mol Pharmacol.

[B32] Sonner JM, Gong D, Eger EI (2000). Naturally occurring variability in anesthetic potency among inbred mouse strains. Anesth Analg.

[B33] Kissin I, Morgan PL, Smith LR (1983). Anesthetic potencies of isoflurane, halothane, and diethyl ether for various end points of anesthesia. Anesthesiology.

[B34] Flood P, Sonner JM, Gong D, Coates KM (2002). Heteromeric nicotinic inhibition by isoflurane does not mediate MAC or loss of righting reflex. Anesthesiology.

[B35] Kinugawa S, Wang Z, Kaminski PM, Wolin MS, Edwards JG, Kaley G, Hintze TH (2005). Limited exercise capacity in heterozygous manganese superoxide dismutase gene-knockout mice: roles of superoxide anion and nitric oxide. Circulation.

[B36] Kline DD, Yang T, Huang PL, Prabhakar NR (1998). Altered respiratory responses to hypoxia in mutant mice deficient in neuronal nitric oxide synthase. J Physiol.

[B37] Sessler DI (2000). Perioperative heat balance. Anesthesiology.

